# Comparison of the Clinical Efficacy and Adverse Events between Intravesical Injections of Platelet-Rich Plasma and Botulinum Toxin A for the Treatment of Interstitial Cystitis Refractory to Conventional Treatment

**DOI:** 10.3390/toxins15020121

**Published:** 2023-02-02

**Authors:** Jia-Fong Jhang, Wan-Ru Yu, Hann-Chorng Kuo

**Affiliations:** Department of Urology, Hualien Tzu Chi Hospital, Buddhist Tzu Chi Medical Foundation, Tzu Chi University, Hualien 970, Taiwan

**Keywords:** botox, platelet-rich plasma, interstitial cystitis, intravesical injection

## Abstract

Background: Intravesical injection of Botulinum toxin A (BoNT-A) and platelet-rich plasma (PRP) have been reported to alleviate bladder pain and decrease nocturia in patients with refractory interstitial cystitis/bladder pain syndrome (IC/BPS). Both treatments are novel and there has no comparison between them. This study compared the therapeutic effects and adverse events between IC/BPS patients receiving PRP or BoNT-A injections. Materials and Methods: This study retrospectively analyzed female patients with IC/BPS who were refractory to conventional treatment and received BoNT-A (*n* = 26) or PRP (*n* = 30) injections within the previous two years. Patients were arbitrarily treated with four monthly injections of PRP or a single injection of 100 U of BoNT-A. All injections were followed by cystoscopic hydrodistention. The primary endpoint was the global response assessment (GRA), and secondary endpoints were changes in the O’Leary-Sant IC symptom score, visual analog score (VAS) of bladder pain, voiding diary, and uroflow measures from baseline to six months after the first injection day. Results: The baseline demographics revealed no significant difference between groups. The GRA at one, three, and six months was similar between groups. A significant improvement in IC symptom scores was noted in both groups. Although VAS was significantly improved in overall patients, no significant difference was noted between the PRP and BoNT-A groups at 6 months. Only half of the study cohort had a GRA ≥2 at six months. An increase in the post-void residual was noted one month after the BoNT-A injection, but there was no difference between groups at three and six months. More patients reported dysuria (19.2% vs. 3.3%, *p* = 0.086) and urinary tract infection (UTI, 15.4% vs. 0%, *p* = 0.041) after BoNT-A injection than after the PRP injections. The time from the first injection to receiving alternative treatment was similar between groups. Conclusion: Both intravesical PRP and BoNT-A injections have similar efficacy in IC symptom improvement. However, only half of the study cohort had a GRA of ≥2 at the six-month follow-up BoNT-A injection carries a potential risk of UTI after treatment.

## 1. Introduction

Interstitial cystitis/bladder pain syndrome (IC/BPS) is a chronic bladder inflammatory disease characterized by bladder pain and frequency urgency. Current treatments using pain killer or anti-inflammatory medications cannot completely eradicate symptoms and increase bladder capacity [1]. Several intravesical or oral medications, such as pentosanpolysulphate, amitryptynine, and cyclosporin have been tried, but their therapeutic efficacy has been proven ineffective [2,3,4,5,6]. The lack of a reliable effective therapy for IC/BPS may be related to its poorly understood pathophysiology. One of the most common findings in bladder mucosal biopsies from patients with IC/BPS is denudation or thinning of the bladder epithelium, suggesting an altered regulation of urothelial homeostasis [7,8]. Other bladder abnormalities include an increased nerve fiber density, inflammatory cell infiltrations, and noxious sensory receptor immunoreactivity [9]. Although investigations on this topic have been enthusiastically performed, the etiology of IC/BPS remains unknown. Treatment based on a single pathophysiology might not be sufficient to solve the underlying pathology of IC/BPS.

Intravesical botulinum toxin A (BoNT-A) and platelet-rich plasma (PRP) injections are novel treatments for IC/BPS refractory to conventional therapies. BoNT-A can reduce the release of acetylcholine and inflammation-related neuropeptides from nerve terminals [10]. The BoNT-A injection can eliminate noxious stimulation and reduce bladder suburothelial inflammation, thus improving urothelial regeneration and IC/BPS symptoms. PRP can also produce new inflammation and override unresolved inflammation in IC/BPS bladders [11]. Through repeated injections, the bladder inflammation is eliminated and regeneration of the defective urothelium is improved, resulting in a healed bladder urothelium with adequate barrier function and a reduction in bladder pain [12]. Although there is solid evidence that BoNT-A improves the IC/BPS condition, a decrease in detrusor contractility following treatment may contribute to a poor response to the BoNT-A injection [13]. On the other hand, the PRP injection does not have such adverse events. However, the effect of PRP on inflammation might be less remarkable than that of BoNT-A; frequent monthly PRP injections are necessary to achieve a therapeutic efficacy similar to that of BoNT-A on IC/BPS [14].

Patients with chronic IC/BPS usually cannot be successfully treated with conventional medical or intravesical treatments. Although previous clinical trials provided evidence that intravesical BoNT-A or PRP injections were effective [11,12,13], the true clinical efficacy and patients’ satisfaction have not been reported in real life practice. To date, there has been no head-to-head comparison between these two treatment modalities. Therefore, we compared these two novel therapies to establish which treatment provides superior treatment efficacy and safety.

## 2. Results

Among the 56 female patients included in the study, 30 received four monthly PRP injections and 26 received a single BoNT-A injection. Patients’ baseline demographics, including symptom score, voiding diary data, uroflowmetry data, findings of cystoscopic hydrodistention, and duration of IC/BPS symptoms are listed in Table 1. There was no significant difference between the two treatment groups; however, a higher VAS score for bladder pain was perceived at baseline in the BoNT-A group.

Table 2 shows the changes in IC symptom score, VAS scores for bladder pain, and GRA from baseline to all follow-up points between groups. The improvement in ICSI and ICPI from baseline to each time-point was significant in both groups, but no significant difference was noted between groups. The change in the VAS score after treatment was significant only in the BoNT-A group; however, the change in VAS score from baseline to the follow-up time points was not significant between groups. Interestingly, the GRA was significantly improved only in the PRP group.

Table 3 shows the differences in voiding and uroflowmetry parameters between the PRP and BoNT-A groups. Although nocturia and FBC were improved in the PRP group and frequency was improved at the one-month follow-up in the BoNT-A group, there was no significant difference all follow-up time points in the BoNT-A group; however, the change in post-void residual (PVR) was only significantly higher in the BoNT-A group at one month. We found the GRA increased with repeated monthly injections of PRP, whereas the GRA was mildly improved immediately after BoNT-A injection but improved to a level similar to that of PRP at six months. Nevertheless, more patients in the BoNT-A group complained of dysuria after treatment, and a significantly higher rate of urinary tract infection (UTI) (15.4% vs. none) developed after the BoNT-A injection compared with that of patients receiving the PRP injection (Table 4).

**Figure 1:**
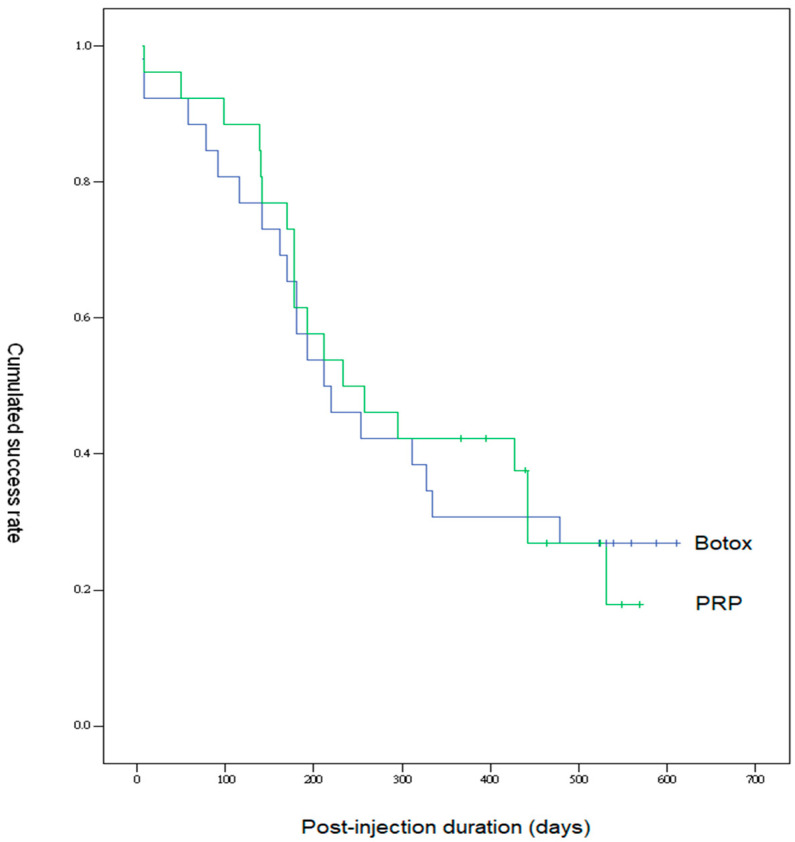
Survival curves of patients with IC/BPS who received either intravesical PRP or BoNT-A injections. Curves from the initial treatment day to the time in which they received additional treatment.

Figure 1 shows the cumulative success rate curve after intravesical PRP or BoNT-A injection. Approximately 50% of patients in each group received an additional bladder therapy for bothersome symptoms after six months. There was no significant difference in the successful curves with time between groups.

## 3. Discussion

This study demonstrated that intravesical injections of either PRP or BoNT-A are safe and effective in IC/BPS symptoms improvement without significantly increasing the PVR. Bladder pain reduction was not significant between groups at 6 months. Patients who received PRP injections did not experience UTI or dysuria after treatment. However, only half of the study cohort had a GRA of ≥2 at the six-month follow-up, and the remaining patients sought additional bladder therapy to improve symptoms. Although the therapeutic efficacy for IC/BPS at 6 months was similar between PRP and BoNT-A, the patients who received the BoNT-A injection had a greater potential risk for UTI after treatment.

The pathophysiology of IC/BPS is complicated and not completely understood. The recent IC Data Base study noted that loss of epithelial integrity is a predominant histopathologic finding in patients with IC/BPS. The epithelial damage may precede other histopathologic findings, such as suburothelial inflammation and sensory nervous activation in the bladder wall, leading to increases in the sensation of bladder fullness and pain in response to bladder inflammation [9,15]. The inflammation of IC/BPS might increase the sensory neuropeptides release from the suburothelial nervous network and integrate the signal transmission from urothelium to the detrusor muscles. In animal models of chemical cystitis or human studies of IC/BPS, detrusor injection of BoNT-A has been shown facilitate an increase in bladder capacity and relief of bladder pain [16,17]. Inhibition of sensory fiber neuroplasticity and inflammation in the suburothelial space by BoNT-A injections provides good therapeutic efficacy in patients with IC/BPS [18]. However, a single BoNT-A injection might not be adequate to provide long-term durability for IC/BPS. Our previous study also demonstrated that four consecutive BoNT-A injections provide a longer therapeutic duration than that of treatment with fewer injections [19].

An intravesical injection of 100–200 U of BoNT-A followed by cystoscopic hydrodistention has been reported effective in decreasing bladder pain and nocturia in patients with IC/BPS [20]. A randomized, double-blind clinical trial demonstrated that 100 U of BoNT-A is safe and effective for treatment of IC/BPS [21]. Patients who received BoNT-A treatment had a significantly greater improvement in bladder pain reduction and bladder capacity increase than that of those in the placebo group. The therapeutic effects of BoNT-A on IC/BPS are result from inhibition of the noxious neurotransmitter releases [17]. Based on the above clinical and basic science evidence, BoNT-A injection was listed as the fourth-line therapeutic option in the treatment of IC/BPS in the AUA guidelines [22].

Although the BoNT-A injection seems promising for treating symptoms of IC/PBS, long-term results have not revealed a successful outcome [21]. The limited successful result is possibly due to inadequate control of chronic inflammation inside the urinary bladder [17]. Repeated intravesical BoNT-A injections were recently performed for patients with refractory IC/PBS, and the therapeutic effects appear promising. Approximately 70% of patients with non-ulcer type IC/PBS may benefit from repeated BoNT-A injections every six months [23]. A previous immunohistochemistry study also confirmed the reduction in inflammatory biomarkers and pro-apoptotic proteins, such as Bax and Bad expressions, after repeated BoNT-A injections. Furthermore, the adhesive protein E-cadherin and junction protein zonula occludens were increased after repeat BoNT-A injections. These immunohistochemistry changes correlated well with the improvement in clinical symptoms [24].

Autologous PRP is growing in the treatment to augment wound healing, fasten the recovery speed of muscle and joint injuries, and enhance surgical repair recovery [25]. PRP is extremely rich in several essential growth factors and cytokines, which regulate tissue reconstruction, and has been studied extensively among trauma patients and trauma experimental models [26]. Tissue regeneration can be improved by the local application of autologous bone marrow derived progenitor cells and PRP. In addition, PRP eliminates neuropathic pain primarily by platelet- and stem cell-released factors. These factors initiate the complex cascade of wound healing events, starting with the induction of enhanced inflammation and its complete resolution, including tissue remodeling, wound repair, and axon regeneration, resulting in neuropathic pain elimination; some of these same factors also act directly on neurons to promote axon regeneration, thereby eliminating neuropathic pain [27].

PRP injecting into the bladder wall could initiate the wound healing process, induce a new inflammation, complete the wound healing, resolve previous inflammation, and promote the relief of neuropathic pain. The cytokines and growth factors released from PRP could induce a new inflammation, which might override the residual inflammation, and increase tissue regeneration [25]. Our previous clinical trial demonstrated that multiple low-dose PRP injections were effective in patients with IC/BPS. The PRP injections could effectively decrease IC symptoms and VAS bladder pain scores from 3.38 ± 2.89 at baseline to 1.10 ± 1.85 at 3 months after PRP injections [28]. The elevated urinary cytokines and inflammatory proteins could also be reduced after repeated PRP injections [29]. Moreover, the ultrastructural deficits in IC/BPS urothelial could also recovered after intravesical PRP injections [30].

Although this was not a head-to-head randomized trial, the results of this comparative case series study reveal that PRP and BoNT-A are equally effective in reducing IC symptoms. Based on the therapeutic mechanism of PRP and BoNT-A on IC/BPS, either treatment can reduce chronic inflammation and improve urothelial regeneration [12]. Therefore, IC symptoms and urinary inflammatory cytokines can decrease after treatment [29]. However, BoNT-A has a more potent inhibitory effect on the release of noxious inflammatory neuropeptides and could better reduce bladder pain, compared to the effect of the PRP injection. On the other hand, BoNT-A reduces detrusor contractility by inhibiting acetylcholine from efferent nerves; thus, a larger PVR may develop after treatment [17]. Nevertheless, the inhibitory effect of BoNT-A on detrusor contractility in IC/BPS bladders was less than that observed in patients with overactive bladders [31]. Although the increased PVR is not clinically significant, this adverse event might lead to a higher rate of difficult urination and increase the risk of UTI after BoNT-A injections.

Both PRP and BoNT-A treatments are novel and currently off-labeled therapies for IC/BPS refractory to conventional therapy. The long-term efficacy has not been well investigated. Although fewer adverse events were reported following treatment with PRP than with BoNT-A, the therapeutic effect of PRP on chronic inflammation and for promoting recovery of defective urothelium was limited. As shown in this study, the GRA increased with the increasing number of PRP injections, suggesting the therapeutic efficacy needs an adequate PRP dose to achieve a therapeutic level. Therefore, repeated intravesical PRP injections every month are necessary to achieve a therapeutic efficacy similar to that of BoNT-A at six months. However, repeated monthly PRP injection treatment requires frequent anesthesia, which might place a tremendous burden on the patients. Four monthly injections might also increase the complications related with bladder injections, including hematuria and UTI. Although therapy with a single high-dose injection of PRP has been attempted, the efficacy on symptom improvement was inferior to that of four monthly low-dose PRP injections [14]. In addition, the economic burden of frequent admission, anesthesia, and the cost of preparing the PRP might be much higher than that of a single BoNT-A injection within a six-month treatment period. Therefore, although the BoNT-A injection bears a greater potential for detrusor underactivity and UTI, repeated BoNT-A injections every six months have the advantage of greater bladder pain improvement and less of an anesthesia burden, with the same efficacy as that of PRP treatment. Single BoNT-A injection only provides short-term therapeutic effect, whereas repeat injections provide a higher success rate in long-term follow-up. Before recommending either treatment to patients with refractory IC/BPS, patients should be thoroughly informed as to the efficacy and potential adverse events of both treatments to allow for shared decision-making.

Limitations of this study are small case numbers, the lack of a control arm, and the non-randomization of the study design. The treatment option was based on patients’ choice after informing advantage and disadvantages of the treatment outcome and potential adverse events. Because all patients were chronic IC/BPS and had received many different conventional therapies, they might have had high expectations to BoNT-A or PRP injection. Further, all patients were not selected with a strict inclusion criterion as in the clinical trials but were diagnosed according to their present symptoms and past history. Therefore, the patients in this retrospective study are highly heterogeneous with varying severity of disease. Finally, although a GRA ≥2 was reported in around 50% of patients treated with BoNT-A or PRP and the ICSI and ICPI also showed improvement, the decrease in bladder pain VAS was limited. The higher VAS in the baseline of the BoNT-A group could have resulted in bias in the VAS reduction between PRP and BoNT-A group. Because this was a retrospective analysis, patients were informed of the advantages and disadvantages of treatment at baseline, and patients were allowed to choose treatment; therefore, more patients who had a higher bladder pain VAS might have chosen the BoNT-A injection. Although a significant reduction in bladder pain VAS was observed in the BoNT-A group, the difference of VAS changes with time between PRP and BoNT-A groups was not significant. Nevertheless, the results of this study may reflect the treatment outcome of BoNT-A and PRP injection in a real-life practice.

In clinical trials for functional urology such as in IC/BPS, OAB, or LUTS, the subjective primary endpoint and objective secondary endpoints are usually not equally improved. Although the primary endpoint can reach a significant result, a placebo effect of up to 30% can usually be found in clinical trial. In this study we found the treatment success was limited in GRA improvement and the other objective variables such as voiding diary parameters, bladder volume increment, and bladder pain reduction were very mild. These results bring a message that, in real-life practice, treatment with a single small dose of BoNT-A or PRP might not be as successful as that in clinical trials. Nevertheless, the safety endpoints also revealed that both treatments were safe and tolerable. With repeated injections of BoNT-A or PRP, the treatment success might be improved. In the future, randomized controlled trials with different doses might be necessary and a search for prognostic factors could help urologists select IC/BPS patients for a good response to PRP or BoNT-A injections.

## 4. Conclusions

The results of this comparative case series study revealed that intravesical injections of either PRP or BoNT-A were safe and effective for improving IC/BPS symptoms without significantly increasing the PVR, but the bladder pain reduction was limited. Only half of the study cohort had a GRA ≥2 at six months. Repeat treatment of PRP or BoNT-A would be necessary to achieve long-term success. The BoNT-A injection carries a potential risk of UTI after treatment compared with that of the PRP injection.

## 5. Materials and Methods

A total of 56 female patients with confirmed IC/BPS who had failed conventional treatments received either a single intravesical BoNT-A 100 U injection (*n* = 26) or four monthly PRP injections (*n* = 30) within the two previous years. The IC/BPS was diagnosed according to the characteristic symptoms and cystoscopic findings after hydrodistention under anesthesia [32]. All patients had previously received at least two types of treatment modalities, including oral medication and intravesical treatment in recent years, with persistent bothersome symptoms and bladder pain. All potential patients received detailed urological examinations and were excluded if the diagnosis failed to meet the criteria of the National Institute of Diabetes and Digestive and Kidney Diseases (NIDDK) [33]. The patients were not randomly allocated to the BoNT-A or PRP group but were treated based on their choice. This study was approved by the Research Committee of Hualien Tzu Chi Hospital (IRB: 111-257-B, dated 15 December 2022). The requirement for informed consent was waived because this study was a retrospective analysis of data.

Patients’ preferred treatment was scheduled after a discussion regarding the advantages and potential adverse effects of each treatment type. They were treated with either (1) an intravesical injection of 10 mL PRP (which was extracted from 50 mL of patient’s blood) at 20 sites every month for four months, or (2) one intravesical dose of BoNT-A 100 U at 20 injection sites. Both treatments were followed by cystoscopic hydrodistention in the operation room. The primary endpoint was the global response assessment (GRA) recorded at six months after the first treatment day. The GRA scale included 6 items: worsening of symptoms (less than 0%), no change (0%), mild improvement (less than 50%), moderate improvement (50–75%), marked improvement (more than 75%), and completely cured (100%) [34].

All patients were asked to maintain a three-day voiding diary at baseline for recording the functional bladder capacity (FBC), urinary frequency, and nocturia. The IC symptoms were assessed by the O’Leary-Sant symptom score (OSS), including IC symptom index (ICSI) and IC problem index (ICPI) [35]. The bladder painwas scored by a self-assessed 10-point visual analog scale (VAS) system [36]. A videourodynamic study (VUDS) and a potassium chloride sensitivity test were routinely performed to exclude patients with detrusor overactivity or bladder outlet obstruction. All patients were informed of potential complications related to anesthesia and bladder injections, such as gross hematuria, micturition pain, and increased bladder pain after PRP or BoNT-A injection. Patients were also informed that the BoNT-A injection could lead to a greater reduction in bladder pain, and both BoNT-A and PRP injection could reduce the frequency episodes after treatment. However, BoNT-A injection might have adverse events of difficulty in urination, increased PVR, and UTI after injection, which had not been observed in our previous clinical trials of PRP injection.

The VUDS was performed at baseline and time-points after intravesical injections. The VUDS was performed with an infusion rate of 20 mL/min, and the procedures and terminology were according to the International Continence Society recommendations [37]. After the VUDS study, a potassium chloride (KCl) test using 0.4 M KCl solution infused into the bladder was performed. A positive KCl test was considered when a patient perceived a painful (VAS score of ≥2) or urgency sensation (urgency severity score increased by ≥1) [38].

PRP was prepared according to previously reported standard procedures [17]. In brief, 50 mL of whole blood was withdrawn at the same day of treatment. The blood was sent to the central laboratory and the blood was centrifuged twice with a slow spin (190× *g*, 20 min, <20 °C) and fast spin (2000× *g*, 20 min, <20 °C) of the supernatant plasma containing platelets. The lower third of the tube comprised PRP, and the upper part was platelet poor plasma (PPP). The platelet pellets were added to the plasma to obtain the desired concentration of PRP. In this study, 6 mL of sterile PRP was obtained. We sent 1 mL of PRP for culture and platelet count. The remaining 5 mL of PRP was used for intravesical injection. A total of 20 suburothelial injections of the PRP solution were performed, with 0.25 mL of PRP at each site, using a 23-gauge needle and a rigid injection instrument (22 Fr, Richard Wolf, and Knittlingen, Germany). The injection was approximately 1 mm in depth into the suburothelium equally distributed at posterior and lateral bladder walls, Cystoscopic hydrodistention was performed immediately after PRP injection to activate the injected platelets and determine the maximal bladder capacity (MBC). The PRP injection procedure was repeated every month for four months. A total of 10 mL of sterile normal saline was used to dissolve the BoNT-A powder to a concentration of 5 U at each site. The BoNT-A solution was gently shaken and then slowly withdrawn and injected at 20 well-distributed sites at posterior and lateral bladder wall followed by cystoscopic hydrodistention as previously reported [17]. After the PRP or BoNT-A injections, a urethral catheter was indwelled overnight, and oral antibiotics were taken for three days.All patients were followed up at one, three, and six months after the first injection day. Urinalysis was routinely checked at each time point and UTI was considered if patients had bladder pain or micturition pain and a white blood cell count >10/high power field in urinalysis.

The three-day voiding diary data (including FBC, daily frequency and nocturia episodes), ICSI, ICPI symptom score, and bladder pain VAS were recorded at baseline (first PRP and BoNT-A injection), and at one, three (fourth PRP injection), and six (three months after fourth PRP, and six months after BoNT-A injection) months. At the one-, three-, and six-month (primary endpoint) follow-up, patients reported any improvement in IC symptoms and adverse events were recorded. An excellent treatment outcome was considered when patients reported a GRA of ≥2 or no bladder pain (VAS = 0). The outcome was considered improved if there was improvement in the GRA by = 1 or the pain VAS score reduced by two or more and there was at least a 25% decrease in urinary frequency and nocturia. Patients with excellent and improved results were considered as having a successful treatment. After the primary endpoint assessment, the patients were continuously followed up with medications, such as anti-inflammatory agents and pain killers only. If the patient’s IC symptoms exacerbated and they requested bladder therapy, the duration from the first injection day to the consecutive bladder therapy was recorded as the effective duration.

The data of voiding diary, VUDS parameters, symptom score, and bladder pain VAS score at baseline, one, three, and six months after the first injection day were compared in each group. A successful result was assessed by a self-reported improvement in GRA and pain VAS score. The data were presented as the mean ± standard deviation. Comparisons between PRP and BoNT-A groups were analyzed by the Student’s t-test to compare numerical data, and the Chi-square test for categorical data. Cumulative success rate was also calculated using Kaplan–Meier survival curves to compare the success rates from the initial treatment day to receiving additional treatment between groups. All statistical analyses were performed using the statistical package SPSS for Windows (Version 12, SPSS, Chicago, IL, USA). A *p*-value of less than 0.05 was considered statistically significant.

## Figures and Tables

**Table 1:** Baseline demographics of patients with interstitial cystitis who underwent intravesical platelet-rich plasma or botulinum toxin A injection.

	PRP (*n* = 30)	BoNT-A (*n* = 26)	*p*-Value
Age (years)	52.57 ± 11.08	49.19 ± 17.03	0.392
ICSI	12.57 ± 4.43	13.15 ± 4.15	0.613
ICPI	11.63 ± 3.21	12.23 ± 3.23	0.492
VAS	3.57 ± 3.14	5.35 ± 3.02	0.036
Frequency	11.70 ± 6.39	13.95 ± 8.35	0.260
Nocturia	3.28 ± 1.59	3.77 ± 5.33	0.651
FBC (mL)	255.83 ± 126.74	264.16 ± 103.70	0.793
Qmax (mL/s)	13.17 ± 7.01	16.54 ± 9.60	0.136
Volume (mL)	192.43 ± 109.65	238.38 ± 112.70	0.128
PVR (mL)	22.97 ± 45.04	21.73 ± 35.38	0.911
MBC (mL)	768.33 ± 173.94	728.40 ± 219.94	0.455
Glomerulation	1.37 ± 0.89	1.42 ± 0.93	0.841
Ulcer	0 (0.0%)	2 (7.7%)	0.211
Duration (years)	10.27 ± 8.85	8.92 ± 10.26	0.601

Abbreviations: PRP: platelet-rich plasma, BoNT-A: botulinum toxin A, ICSI: interstitial cystitis symptom index, ICPI: interstitial cystitis problem index, VAS: visual analog score, FBC: functional bladder capacity, Qmax: maximum flow rate, PVR: post-void residual, MBC: maximal bladder capacity.

**Table 2:** Changes in symptom scores and the global response assessment after intravesical PRP or BoNT-A injection for patients with IC/BPS.

		All (*n* = 56)	PRP (*n* = 30)	BoNT-A (*n* = 26)	*p*-Value
ICSI	BL	12.84 ± 4.27	12.57 ± 4.43	13.15 ± 4.15	
	1M	9.95 ± 4.35 *	9.43 ± 3.32 *	10.54 ± 5.30 *	0.616
	3M	9.55 ± 4.16 *	9.57 ± 3.66 *	9.54 ± 4.75 *	0.598
	6M	9.00 ± 4.21 *	8.33 ± 4.40 *	9.77 ± 3.92 *	0.410
ICPI	BL	11.91 ± 3.20	11.63 ± 3.21	12.23 ± 3.23	
	1M	9.20 ± 4.26 *	8.93 ± 3.54 *	9.50 ± 5.02 *	0.975
	3M	8.50 ± 3.69 *	8.87 ± 3.21 *	8.08 ± 4.20 *	0.137
	6M	8.73 ± 3.97 *	8.60 ± 4.06 *	8.88 ± 3.94 *	0.781
VAS	BL	4.39 ± 3.18	3.57 ± 3.14	5.35 ± 3.02	
	1M	4.27 ± 3.11	4.00 ± 3.22	4.58 ± 3.02	0.062
	3M	3.38 ± 2.90 *	3.20 ± 3.01	3.58 ± 2.80 *	0.068
	6M	3.66 ± 3.06 *	3.17 ± 3.18	4.23 ± 2.87 *	0.239
GRA	1M	0.84 ± 1.16	0.73 ± 1.08	0.96 ± 1.25	
	3M	0.89 ± 1.65	0.83 ± 1.58	0.96 ± 1.75	0.840
	6M	1.36 ± 1.55 *	1.53 ± 1.20 *	1.15 ± 1.89	0.227

* *p* < 0.05 compared with baseline data, Abbreviations: GRA: global response assessment, PRP: platelet-rich plasma, BoNT-A: botulinum toxin A, ICSI: interstitial cystitis symptom index, ICPI: interstitial cystitis problem index, VAS: visual analog score.

**Table 3:** Changes in voiding and uroflow measurements after intravesical PRP or BoNT-A injection for patients with IC/BPS.

		All (*n*= 56)	PRP (*n*= 30)	BoNT-A (*n* = 26)	*p*-Value
Frequency	BL	12.74 ± 7.38	11.70 ± 6.39	13.95 ± 8.35	
	1M	11.40 ± 5.83	10.88 ± 4.98	11.99 ± 6.73 *	0.475
	3M	12.09 ± 6.48	10.77 ± 4.18	13.62 ± 8.21	0.729
	6M	11.72 ± 5.63	10.95 ± 4.08	12.60 ± 6.99	0.694
Nocturia	BL	3.51 ± 3.78	3.28 ± 1.59	3.77 ± 5.33	
	1M	2.74 ± 3.00	2.55 ± 1.50 *	2.97 ± 4.13	0.936
	3M	2.63 ± 3.19 *	2.22 ± 1.42 *	309 ± 4.43	0.484
	6M	2.57 ± 3.06 *	2.35 ± 1.80 *	2.83 ± 4.08	0.977
FBC	BL	259 ± 116	256 ± 127	264 ± 104	
	1M	283 ± 123 *	276 ± 124	292 ± 125	0.783
	3M	299 ± 126 *	303 ± 135 *	295 ± 118	0.935
	6M	314 ± 138 *	305 ± 136 *	325 ± 142	0.465
Qmax	BL	14.73 ± 8.41	13.17 ± 7.01	16.54 ± 9.60	
	1M	14.74 ± 9.61	13.76 ± 8.02	15.88 ± 11.23	0.548
	3M	16.04 ± 9.49	16.87 ± 10.48	15.10 ± 8.29	0.043
	6M	15.98 ± 10.08	15.93 ± 10.80	16.03 ± 9.40	0.188
Volume	BL	214 ± 112	192 ± 110	238 ± 113	
	1M	207 ± 132	196 ± 123	218 ± 143	0.425
	3M	212 ± 106	199 ± 93.9	227 ± 118	0.512
	6M	232 ± 114	218 ± 114	248 ± 115	0.608
PVR	BL	22.39 ± 40.49	22.97 ± 45.04	21.73 ± 35.38	
	1M	39.88 ± 56.78 *	25.97 ± 37.95	55.92 ± 70.16 *	0.045
	3M	34.46 ± 50.64	21.70 ± 27.06	49.19 ± 66.11 *	0.070
	6M	43.79 ± 78.38 *	40.97 ± 88.59	47.04 ± 66.26 *	0.735

* *p* < 0.05 compared with baseline data, Abbreviations: PRP: platelet-rich plasma, BoNT-A: botulinum toxin A, FBC: functional bladder capacity, Qmax: maximum flow rate, PVR: post-void residual.

**Table 4:** Changes in the global response assessment and adverse events after intravesical PRP or BoNT-A injection for patients with IC/BPS.

	PRP (*n* = 30)	BoNT-A (*n* = 26)	*p*-Value
1M GRA ≥ 2	5 (16.7%)	9 (34.6%)	0.122
3M GRA ≥ 2	12 (40.0%)	10 (38.5%)	0.906
6M GRA ≥ 2	14 (46.7%)	13 (50.0%)	0.803
AE-Dysuria	1 (3.3%)	5 (19.2%)	0.086
AE-UTI	0 (0.0%)	4 (15.4%)	0.041

Abbreviations: GRA: global response assessment, PRP: platelet-rich plasma, BoNT-A: botulinum toxin A, AE: adverse event, UTI: urinary tract infection.

## Data Availability

Data can be obtained with permission of the corresponding author.
